# Secundum Naturam Vivere: Stoic Thoughts of Greco-Roman Antiquity on Nature and Their Relation to the Concepts of Sustainability, Frugality, and Environmental Protection in the Anthropocene

**DOI:** 10.1007/s40926-023-00233-8

**Published:** 2023-03-30

**Authors:** Hendrik Müller

**Affiliations:** Hochschule Fresenius Fachbereich Onlineplus: Hochschule Fresenius online plus GmbH, Berlin, Germany

**Keywords:** Environmental humanities, Stoa, Sustainability, Frugality, Anthropocene

## Abstract

This paper wants to shed light on the way the philosophical school of Stoicsm in Greco-Roman antiquity has dealt with the relationship of men and nature by pointing out to some of the key texts in which these issues are mentioned. Although the modern concept of sustainability or environmental protection did not really exist in antiquity, the Stoa was convinced that individual decisions had a direct impact on this world. Following the concept of environmental humanities, the ancient texts and authors are collected as historical ideas of the multifaceted interactions between nature and men that can be fruitfully mirrored with the arguments of the current Anthropocene discourse and its focus on (post)industrialism. By doing so we might come across helpful approaches deeply rooted in our cultural heritage that we could possibly adopt and find practical answers for our age in terms of individual behaviour as well as management decisions to face the ecological and social challenges ahead.

The actual discourse, both scientific and social, is heavily influenced by the topics of sustainability and environmental protection. It seems one of the key insights of the Anthropocene epoch (Crutzen 2006) that for far too long mankind has exploited the natural resources of the earth without showing any respect for nature´s diversity. The more the effects of man-made climate change become obvious, the more unsure we are about the consequences of this change, and we are even more undecided about the methods of solving the most pressing problems like rising sea-levels, increasing thermal extremes and weather disasters. Yet, the awareness of the vulnerability of our natural surroundings is not a recent phenomenon. Already in the age of classical antiquity some philosophers and thinkers have dealt with the question, how we humans should handle natural resources, and which impact our behaviour on nature has in the present and could have in the long run. The following paper wants to shed light on the way ancient Stoicism as a philosophical school vibrant in the Hellenistic world and the Roman Empire has dealt with challenges of men’s relationship to nature by pointing out to some of the key texts in which these issues are mentioned. Following the recent development of the methodological development of environmental humanities (Schliephake 2020) the ancient texts are analysed from an actual perspective to find out, in which ways we could possibly adopt their outlook and find practical answers for our modern world and in particular management practices. Yet, it should be clear that the parallels could not be overstressed, as “many of the environmental perils and toxic substances that modernity has created were wholly unknown in the ancient world” (Schliephake 2020, 55), and the proclaimed fear of self-extinction of humankind in total because of our collective behaviour is certainly alien to ancient Greco-Roman thoughts. Yet the reception of the ecology-related aspects of ancient philosophy might help us to get a clearer picture of our cultural heritage when it comes to the dependence between natural history and human actions.

The English terms ‘sustainable’ and ‘sustainability’ appeared for the first time in the Oxford English Dictionary in the second half of the 20th century. But the equivalent terms in the German language (`*Nachhaltigkeit*`), in French (´*durabilité*` and `*durable*`), or even in Dutch (`*duurzaamheid*` and `*duurzaam*`) have been used for centuries (van Zon 2002: 20, 21, 22). Although there are no direct verbal equivalents in the ancient languages of Greek or Latin (Glare 1982), we can record the fact that the Greeks and Romans of antiquity, and even earlier civilizations like the Egyptian or Mesopotamian culture describe environmental problems we would today refer to as problems of ecological degradation or sustainability (respectively the lack of it). As societies which even in their urban centres were much more deeply dependent from agriculture than we are, problems like the loss of fertility of soil were constantly noticeable for the ancients. On the other hand, it is especially the increasing urbanization, often as a direct result of armed conflicts and warfare, that could be named as a specific cause that took its destructive toll on nature. The reciprocal influence and relationship between nature and humankind was clearly recognized by ancient writers, e g the Greek physician Hippocrates in the 4th century BC has observed the “effect of climate on human health, temperament, and intelligence and remarked that civilizations arose in lands of moderate or warm climate with light rainfall, where water supply was a major challenge” (Hughes 1975, 3). But let us get an overview of the mentioned sources to get a better picture how the developments mentioned above were perceived by ancient Greek and Roman writers. Most famously the Athenian philosopher Plato (428/427 − 348/347 BC) as early as in the 4th century BC describes the phenomena of soil erosion (*Critias* 111a-b) and deforestation (*ibd*. 111c) and compares this degradation to former times in which there was an abundance of natural resources. Although scholars have discussed controversially, if Plato blames men or natural courses for these incidents, recent archaeological and geological research seem to support his claims: through the method of pollen analysis researchers have found out that soil erosion in the ancient Mediterranean region has been a result of deforestation that is caused by anthropogenic activities (Scheer 2019, 21; Ferreira et al. 2022). On the one hand such developments are connected to periods of human settlement and the following introduction of agriculture and increasing building activities. On the other hand, especially in Plato`s lifetime, larger amounts of wood were needed to organise the building of ships for the fleet used in the Persian as well as in the Peloponnesian war between the philosopher’s hometown Athens and Sparta. But even in earlier centuries, immense amounts of woods were needed between the 8th and 6th century BC to equip the fleets used by Greek settlers for the colonization of various coasts around the Mediterranean Sea. Likewise, the ever growing metallurgical and ceramic industries were highly dependent on natural resources won from felled trees (Runnels 1995; Meiggs 1982). But the relationship between men and nature is far from one-sided, but highly ambivalent as Plato demonstrates elsewhere in his writings, when he mentions periodic natural catastrophes by which men is exposed to nature or even a victim of natural causes like flooding (*Laws* 677) or by fore and water (*Timaeus* 22c).

Yet, if we skip a couple of centuries into Roman-dominated times and into the first century BC, we find notes of the consolidated conviction that humans are to be valued higher than other creatures and that all things in this world have been created and provided for the sake of men. This anthropocentric view was for instance expressed by the famous orator and politician Marcus Tullius Cicero (106 − 43 BC) and reasserted by the claim that only humans are endowed with reason and speech are and therefore differ from lower animals and their sole urge of self-preservation (Cic. Off. 1.14). But on the other hand, other authors express their doubts about the superiority of humans above nature and their use of it and see this topic much more differentiated. There is even a growing belief that mankind is indeed the largest threat for the vulnerability of nature and that natural resources should not be exploited ruthlessly. Cicero´s contemporary Lucius Iunius Moderatus Columella (died ca. 70 BC) is one of the most prominent Roman authors on agriculture and admonishes in his *De re rustica* to maintain the everlasting youth (Col. 1.1) of the earth. Columella makes it also obvious that natural degradation is caused by human activities like logging, mining or farming which has prompted him to record the correct procedures of agricultural activities in his work.

Another Roman philosopher and scholar on agriculture, Marcus Terentius Varro (116 BC – 27 BC) was convinced that men by care could lessen these evil effects. Varro’s *Res rusticae* is another ancient source that recommends specific methods of sustainable farming (Savio 2011), as it contains the famous phrase that agriculture is a science that can teach us that the land may produce the highest yields in perpetuity (Var. R. 1.3) as one of the earliest definitions of sustainability (Jones 2007; Pretty 2007, 7). At the same time Varro condemns the *luxuria* or extravagance of his contemporaries that for instance is reflected in the agricultural architecture, so that Varro suggests orientating more on the habits of the ancestors (Var. R. 1.13.5). This criticism of luxury also becomes graspable some decades later in the epic poem *De bello civili* by the poet Marcus Annaeus Lucanus (39–65 AD) who addresses the topic for example in the description of the North-African tribe of the Mauri who lived in a state of innocence until the axe of Rome brought them the disruptive force of civilization (Luc. 9, 426 − 30). Such statements are not least also an expression of a longing for the imaginary “good old days” which have been disrupted by the political changes and civil war.

## Impact of Warfare and Technology

As mentioned above warfare undoubtedly already in antiquity had an immediate and obvious impact on nature (Hupy 2008) as in particular the Romans were at war with other nations continuously and therefore had to supply a standing army in several regions over centuries. Their soldiers practically lived off the land, as a supply from home was simply not practicable due to the restricted method of land transport (Goldberg and Findlow 1984, 376). Consequently, not only stored or growing crops of the local population might have been plundered by the soldiers, but also the livestock not infrequently fell victim to the need of the army. But these methods could be also used as an intentional tactic to demoralise the enemy and is described as early as in the 5th century BC for Greek soldiers by Xenophon *(*Mem. 2.1.13*).* Centuries later the Roman historian Publius Cornelius Tacitus (ca. 58 AD- ca. 120 AD) reports identical tactics used by Roman troops in his *Agricola*, in which he recounts the life of his father-in-law Gnaeus Julius Agricola (Tac. *Agr*. 30.5). Warfare in general therefore is a direct cause for the constant exploitation of natural resources, but with the continuous progress of civilization it was by far not the only impact on nature by human action.

In line with the progress of technical development changes in the natural environment are becoming increasingly visible. Roman construction and engineering technology had a deep impact on landscapes throughout the whole Roman Empire, with road networks like the *Via appia* criss-cross like lifelines various regions and countries. But improvements in technology also allowed the actual modification of natural conditions and landscapes as the Greek geographer Strabo (64/63 − 24 BC) who is known for his work *Geographica*, identified the Athenian port Piraeus as a former island (Newton 2011). Another technology that became more and more sophisticated in antiquity and by necessity reinforce natural degradation are mining techniques. The natural philosopher Gaius Plinius Secundus (23–79 AD), for differentiation with his homonymous nephew known as Pliny the Elder, was for some time personally responsible as a *procurator* for the administration of a goldmine in Spain. Pliny later described in his encyclopaedic *Naturalis historia* in detail which kind of mining operations were implemented across the Roman Empire. The most lasting visual impact on the landscape obviously had the practice of ‘hushing’ which involved the building of aqueducts to carry vast amounts of water containing the gold-bearing quartz. The power of the stored water later would then be released, as the mountains finally collapsed, stripping the top soil and the vegetation from the land (Hirt 2010, 33 ff; Healy 1999, 280 f). The exposed gold-rich sediments could be further processed afterwards. This technique is commonly known as “ruina montium” (Plin. HN 33.21), and although some scholars have expressed their doubts abit its practicability, the environmental effects of this ancient practice are still clearly visible at the site of the former largest gold mine of the Roman empire at Las Médulas in modern Spain (cf. Domergue 1987).

Pliny regarded mining operations of this kind as a clear violation of nature and criticized his fellow-countrymen for their *luxuria* that makes them to abuse the natural resources of the earth (Wallace-Hadrill 1990, 86). In the opening remarks to Book 33 of his work Pliny thematises the collected knowledge about metals and minerals, and bitterly concludes that the mining technique are clearly driven by pure greed for riches (HN 33.2). Pliny clearly sees nature in this case defeated by the destructive forces of men, as the miners after the collapse could not event be sure that their search for gold would be successful. In other parts of his work, Pliny criticizes the pollution of rivers and other corruptions of nature (HN 18,3) and emphasizes that the earth pays back what is invested in her (HN 2,155) which has tempted Andrew Hallace-Wadrill to seen in him as a sort of proto-environmentalist (Cf. Wallace-Hadrill, 1990, 85). In summary we can clearly conclude, that already in antiquity the vulnerability of nature was an acknowledged fact and the more human civilizations spread out the more negative effects would become visible and were voiced with a certain concern. Yet, it is inevitable that results in of nature. Views of that kind seemed to increase in the first centuries BC and AD, that mark an era of upheaval between the dying Roman Republic and the emerging and changeable empirical rule, so the worries of Pliny and others that could also be interpreted as a reflection of a political vulnerability and insecurity. Yet these declared ancient doubts are naturally far removed from the existential fears that mankind could face in the 21st century. And although the ancients were obviously likewise exposed to climate change and its consequences like pandemics, as Kyle Harper has demonstrated recently (Cf. Harper 2017), they must be seen as individual voices reflecting an elite culture and could not be taken for granted as general point of view, as Daniela Dueck has demonstrated recently (Cf. Dueck 2021).

## The Treatment of Animals

As a further argument the ancient treatment of animals might serve as evidence for the ruthlessness and brutality of mankind against nature. Especially the *venatio*, the hunting and killing of wild animals in the amphitheatre as an integral part of the gladiatorial games, was heavily criticised or at least controversially discussed by prominent ancient writers. This form of entertainment seems to be introduced to Rome in the 2nd century BC and especially been adapted by the emperors and other officials to satisfy the taste of the urban audience. Augustus famously records with some pride that during his reign (31 BC – 14 AD) he has sponsored 26 of these events, during which no less ca. 3500 animals been killed (Cf. R.Gest.div.Aug. 22).

Yet not all people considered this blood sport as appropriate, starting with Cicero who thought the practice of the games was no apt entertainment for an educated man (Cf. Cic. Fam. 7.13). Similarly, the philosopher Lucius Annaeus Seneca (3BC to 65 AD), condemned the *venationes* for their negative impact on the human soul (cf. Ep. 1, 7). The Greek writer Plutarch (ca. 45–126 AD) is even clearer, comparing the *ludi* with the cruel games of small boys who throw stones at frogs to torture and kill them, just for the sake of fun. In his `*De sollertia animalium*` Plutarch also contradicts the common view of his contemporaries that animals lack intelligence and therefore had no rights. Plutarch „argues that killing animals leads to human insensibility and outlines the process of the decline of human nature” (Jazdzewska 2009/10, 37).

Another Roman author who followed this view closely was Claudius Aelianus (175–235 AD) who in his miscellany *De rerum animalium* or *On the nature of animals* repeatedly unmasks men as the inferior species to and clearly contradicts the Ciceronian view about the superiority of men mentioned above. Following the assumption of the irrationality of animals, Aelian is valuing their achievements and abilities even more highly (Müller-Reineke 2010). As an illustrative example he retells the famous anecdote about the slave Androcles (Cf. Ael. NA 7.48) who took refuge from his master’s cruelty in in a cave, when a lion entered the cave and showed him his swollen paw. By extracting a large thorn from the wound Androcles eased the animal’s pain. Years later the grateful animal could make amends when both met again in a public display in the arena. Instead of attacking Androcles who has been captured and thrown to the wild beasts in the arena, the lion began to caress his old friend; with the result that both animal and human were set free. Aelian reports that the animal was the one who recognised his companion immediately while it took a while until the situation became apparent to Androcles. This example that should prove that memory is also an attribute of animals, in a different account Aelian highlights even more admirable skills by presenting the miraculous story of an elephant that could write Latin and demonstrated in this skill in the Rome (Cf. Ael. NA 2.11). Aelian clearly emphasizes the abilities of certain species as admirable and consciously points out their moral strength that by implication reveals the weakness of humanity relentlessly.

Apart from these moral reservations against the animal hunts, the games in late antiquity quite obviously also endangered the existence of certain species: In the 4th century AD the rhetor Themistius Euphrades (317–388 AD), who was a prominent Constantinopolitan senator, and during his lifetime served as an adviser to several Roman emperors, complained in one his published orations about the fact that elephants in Lybia, lions in Thessaly and hippopotamuses in Egypt were nearly extinct due to their excessive use in the *venationes* in the theatres of the empire and pleads for protective measures (Cf. Them. Or. 10.140).

Although Themistius did not explain clearly what measures of protection were taken to preserve the mentioned species, his remark could mean that “the provincials in the affected areas were officially banned from hunting or capturing them” (Epplett 2002, 228).

## Stoic Principles

Many of the named writers were followers or at least sympathised with the philosophical school of the Stoa whose doctrines were initially taught by Zeno of Citium (ca. 334 – ca. 262 BC) in Athens since 300 BC. Zeno based his philosophical teachings on three separate pillars of logic, ethics and physics of which the last one is particularly of relevance here. Zeno’s cosmological view of the universe was that it operates perfectly and is guided by a logical force that he called φύσις, i.e. nature. Consequently, the “ultimate goal of the virtuous life is to “live in agreement with nature” (Smith 2014, 105). Stoic ideas later spread rapidly among Greek and Roman thinkers respectively. Its core teaching that happiness is gained from living a life of virtue in accordance with nature was coined as the Latin phrase *secundum naturam vivere* in the first century AD by the philosopher Seneca the Younger repeatedly in his epistles (cf. ep. 41,9, 121,3) and became the motto of the Stoic school which in the 2nd century attracted even the Roman emperor Marcus Aurelius (121–180 AD) who wrote down his personal thoughts on Stoicism in his famous *Meditations*. The French philosopher Pierre Hadot, who was convinced that regardless of the large time gap between antiquity and our times that ancient philosophy could still be an inspiration for our spiritual life, has worked intensely on this core text of ancient Stoicism and its underlying teachings of Epictetus (Hadot 2001).

Additionally, since a couple of years, Stoicism and the four Stoic virtues of wisdom, courage, justice, and temperance have underlived a recognisable popular revival.^1^ Some of the modern Stoics understand their way of practising Stoicism as a synonym with vegetarianism or environmentalism, but certainly it is the crucial point of personal responsibility that might be helpful as a guidance for businesses as well as for all of us. Already in antiquity the question whether a person’s actions are predetermined by fate, so that he cannot be held responsible for his actions, was discussed widely, most prominently by Cicero in *De fato* in which he records the arguments of the Stoic Chrysippus of Soli (279 − 206 BC) who became as one of Zeno´s successors head of the Stoic school in Athens and expanded many of the fundamental doctrines of Stoicism. Cicero ultimately declares himself in favour of free will. Therefore, many Stoics in antiquity were politically active and, unlike the rivalling school of Epicureanism, did not retreat from the world. Stoics were also told to use humanity’s unique gift of reason and educate the youth to make this world a better place.

Some of the key Stoic ethical arguments have already been randomly popularized for modern management thought (see footnote 1), yet often without a deeper insight into the overall concept of the ancient philosophical school. Yet a deeper engagement should lead to more than collections of quotations or popular science handouts. The Stoics understood nature as the totality of the cosmos, in which we could participate in a distinctively human way, i.e. in accordance with our own specific nature. One of the central terms of Stoic philosophy therefore is ´oikeiois` that can already be pinned down to the writings of Zeno or Chrysippus. Although the term is hard to translate and has been discussed widely ever since (cf. Klein 2016), we can explain it as a process of appropriation in whose course every creature is at home in nature. But the appropriation is not single sided but likewise requires coming to terms with one’s own nature, which includes one`s dark sides. Only then we could become aware how our human self is “fundamentally embedded both in the structure of nature as a whole and in a web of relationships” (Reydams-Schils 2005, 83). This realisation can lead to a changed attitude towards nature in which we all become aware of our embeddedness in natural processes. And only the acceptance of the destructive forces within us will bring about a change that results in a generalised conviviality of men, animals, and nature overall. In the light of the challenges of our time like the overcoming of the corona-pandemic but far more important the threatening consequences of man-made climate change, a global turnaround seems to be more than reasonable and could be the only chance of mankind to tackle the myriad of challenges ahead.

The concept of ‘oikeiosis’ also implies that Stoic virtue ethics demands a life that makes use of himself for the benefit of society (Usher 2020, 150). Following this thread Whiting et al. (2018) have argued convincingly that “ancient Stoicism offers a compelling alternative to the modern economic paradigm of unlimited growth and the depletion of natural resources and the environmental damage that paradigm has caused” (Usher 2020. 152). They conclude that it might be helpful to add the ancient insights of the Stoic school, i.e. to live in agreement with nature to the ongoing Sustainability discourse in order to rethink not only the individual responsibility of each of us, but also the role of materials and the overall question whether they generate a collective virtue in the end. This approach is far from being anachronistic by the way, as the “throwaway culture often associated with modern consumerism was also present in second and third century Rome” (Whiting et al. 2018). As the authors admonish the question remains “how much material is needed to support virtue and how best to measure the collective advancement towards it, using access to and the quality of material services as a proxy” (Whiting et al. 2018). At this it might be helpful to combine the concept of sustainability with the approach of frugality which not only is an integral part of Stoic doctrine in the Greco-Roman word (Stephens 2018) nit is becoming increasingly popular in the industrialized world. A self-imposed frugal lifestyle can be seen as a deliberate choice for con-scious consumption (Herstatt and Tiwari 2020, 12) and is even likely to become a global trend on corporate level likewise: also for companies frugal innovations can have a promising potential for the realization of global sustainability goal and “frugality is perceived as an enabler and new perspective to support the primary goal of (corporate) sustainability” (Achtelik et al. 2022).

## Conclusion

So, in conclusion, which insights of ancient Stoicism might help us in the 21st century to tackle the current ecological problems which were certainly worsened by the current coronavirus disease outbreak? Although the people in antiquity might not had the same feeling of a global catastrophe and endangerment of the human species, it has become clear that philosophers in particular and other men of letters already more than 2000 years ago sensed environmental problems and undesirable developments of human progress. According to the ancient thinkers, these problems were on the one hand a consequence of the ever growing and flourishing civilization, on the other hand many of them lie within human nature and its tendency to constantly change or even destroy our given environment. Yet, in the light of human ambitions, Seneca in his letters repeatedly admonishes the changeability of fate.^2^ However, nowhere in the ancient sources we find a resolute demand for a change in the use of natural resources. So, it becomes evident that the modern concept of sustainability did not exist in antiquity, but as we have demonstrated it could be easily combined with the core teachings of ancient Stoicism. As the anthropologist Philippe Descola have claimed we might rethink our concept of nature (as opposed to culture) (Descola 2013) as a separate object and must return to a more romantic idea of nature as art and us being an integral part of it (Hadot 2008).

Like people in antiquity, each of us today has to make an individual decision which impact she or he wants to have on this world. This applies to our role as a private person as to that of a business person or a (political) leader. In the end ancient philosophy might help us to understand, that it is not the individual decision for a certain number of travels, a specific diet or the number of goods we consume that counts. Furthermore, it is a responsible and critical mind-set that could save this world and make our lives more sustainable and frugal. If Ugo Bardi has calculated the complex theory of the aptly called “Seneca effect” correctly (Bardi 2017), we should not put too much strain on those natural resources that are already depleted but must inevitably embrace necessary change to avoid an overall collapse - the guiding principles of Stoicism might be a good starting point.

## Footnotes

1 It is simply an impossible task to appropriately document to the ever-growing literature, blogs or podcasts on Stoicism in this paper. In the UK a collaboration between academics and psychotherapists has even founded a not-for-profit company in 2021 that offers free online courses, cf. https://modernstoicism.com/.

2 Seneca ist also name giver to the “Seneca effect”, is a mathematical theory that addresses a class of problems in nature in which decline is faster than growth, cf. Bardi 2017. The phrase is based on the quote “*Fortune is of sluggish growth, but ruin is rapid*” (Sen., Ep, 91.6).
